# The relationship between additional heads of the quadriceps femoris, the vasti muscles, and the patellar ligament

**DOI:** 10.1155/2022/9569101

**Published:** 2022-02-16

**Authors:** Łukasz Olewnik, Kacper Ruzik, Bartłomiej Szewczyk, Michał Podgórski, Paloma Aragonés, Piotr Karauda, R. Shane Tubbs, Jose Ramon Sanudo, Maria Bettencourt Pires, Michał Polguj

**Affiliations:** ^1^Department of Anatomical Dissection and Donation, Medical University of Lodz, Poland; ^2^Polish Mother's Memorial Hospital Research Institute, Lodz, Poland; ^3^Department of Orthopedics Surgery, Hospital Santa Cristina, Madrid, Spain; ^4^Department of Neurosurgery, Tulane University School of Medicine, New Orleans, LA, USA; ^5^Department of Human Anatomy and Embryology, Facultad de Medicina, Universidad Complutense de Madrid, Spain; ^6^Department of Surgery and Human Morphology, Universidade NOVA de Lisboa, Lisbon, Portugal; ^7^Department of Normal and Clinical Anatomy, Chair of Anatomy and Histology, Medical University of Lodz, Poland

## Abstract

**Introduction:**

The quadriceps femoris consists of four muscles: the rectus femoris, vastus medialis, vastus intermedius, and vastus lateralis. However, the effect of additional quadriceps femoris heads on the vasti muscles and patellar ligaments is unknown. The aims of the present study are to determine the relationship between additional quadriceps femoris heads and the vasti muscles and patellar ligaments and to review the morphology of the vastus lateralis, vastus medialis, and vastus intermedius.

**Materials and Methods:**

One hundred and six lower limbs (34 male and 19 female cadavers) fixed in 10% formalin were examined.

**Results:**

On all lower extremities, the vastus lateralis consisted of superficial, intermediate, and deep layers. The vastus medialis, on the other hand, consisted of only the longus and obliquus layers. The quadriceps head had one or more supplementary heads in 106 dissected limbs from 68 cadavers (64.1%). The distal portion of the patella was wider in lower limbs without supplementary heads than in type IA but narrower than in type IIIA. In general, the distal portion of the patella was narrower in specimens with a supplementary head than in those without (19.03 SD 3.18 mm vs. 20.58 SD 2.95 mm, *p* = 0.03817). Other patellar ligament dimensions did not differ significantly.

**Conclusion:**

The quadriceps femoris muscle is characterized by high morphological variability. Occurrence of extra heads is at the level of 64.1%. The vastus lateralis consists of three parts (superficial, intermediate, and deep), and vastus medialis consists of two (longus and oblique).

## 1. Introduction

The quadriceps femoris muscle (QF) is important in locomotion; the rectus femoris (RF) swings the leg forward when a step is taken. During walking or running, quadriceps muscles such as the vastus medialis (VM) stabilize the patella and knee joint [1].

The QF is reported to show little morphological variation, only duplication of the VM or vastus lateralis (VL) in some cases [2, 3]. However, its morphological variability has been increasingly studied in recent years [4–7]. A fifth head of the QF, the tensor vastus intermedius (TVI), has been observed [4–6, 8–11]. Also, recent studies suggest that the QF is not exactly a quadriceps; since cases of six, seven, or eight bellies have been observed, it should be termed a multiceps femoris [7, 12].

The QF is important in sport owing to its potential for injury, which can be painful and debilitating. Strains, tears, and contusions are relatively common and require recovery time [7, 13]. The QF can also be weakened by ACL ruptures and reconstructions, resulting in atrophy of the VM and vastus intermedius (VI) [1, 7, 14, 15].

QF allografts can be used during reconstruction of the posterior cruciate ligament (PCL) [16–18], anterior cruciate ligament (ACL) [19–21], medial patellofemoral ligament (MPFL) [22, 23], or fibular collateral ligament (FCL) [24]. Alternatively, such reconstructions can be performed using the hamstring tendon from the pes anserinus [25, 26].

The aims of the present study are to determine the relationship between additional quadriceps femoris heads and the vasti muscles and patellar ligaments and to review the morphology of the VL, VM and VI.

## 2. Materials and Methods

A preprint has previously been published [27]. One hundred and six lower limbs (34 male and 19 female cadavers) fixed in 10% formalin were examined. The cadavers' mean age at death was 68.4 years (43-93), and the initial group comprised equal numbers of female and male adults. The cadavers were the property of the Department of Anatomical Dissection and the Donors and Dissecting Rooms Center, Universidad Complutense de Madrid, Spain, following donation to the university anatomy program. Lower limbs with evidence of surgical intervention in the dissected area were excluded.

The lower limb was positioned in the supine position on the dissection table. First, the hip joint capsule was resected and the inguinal ligament identified. Following this, all femoral branches were dissected. The sartorius and RF muscles were transected in the middle of the muscle belly and lifted to optimize deep vasti view. Each of the vasti bellies was dissected to reveal the origin and insertion of each muscle belly. Finally, the tendons were dissected to identify potential additional tendon bands. After thorough cleansing of vasti muscles, it was checked carefully for additional heads. If found, they were thoroughly cleaned along with the tendons. Then, the appropriate photographic documentation was mocked [7].

### 2.1. Morphometric Measurements and Classification

The types were distinguished on the basis of the classification introduced by Olewnik et al. [7], and the measurements and morphometrics and relationships between the individual parts of the QF and the additional heads were assessed:

1. Lengths of the bellies of the vastus lateralis, vastus medialis, and vastus intermedius muscles and accessory heads of the quadriceps femoris
2. Width/thickness at the musculotendinous junction of the vasti muscles and accessory heads of the quadriceps femoris
3. Length of the vastus lateralis, vastus medialis, and vastus intermedius tendons and accessory heads of the quadriceps femoris
4. Patellar ligament length and patellar ligament widths and thicknesses at origin, middle part, and insertion

An electronic digital caliper was used for all measurements (Mitutoyo Corporation, Kawasaki-shi, Kanagawa, Japan), and each measurement was performed twice with an accuracy of up to 0.1 mm.

### 2.2. Statistical Analysis

Statistica v. 13 software (StatSoft, Cracow, Poland) was used for statistical analyses. A *p* value < 0.05 was considered significant.

The results are presented as means and standard deviations unless stated otherwise. The normality of the continuous data was checked with the Shapiro-Wilk test. As the data were not normally distributed, the anthropometric measurements were compared between sexes using the Mann-Whitney test and between body sides using the Wilcoxon signed rank test. The Kruskal-Wallis ANOVA was used to compare these parameters between proximal attachments types.

## 3. Results

### 3.1. Additional Heads of the Quadriceps Femoris

**Figure 1:**
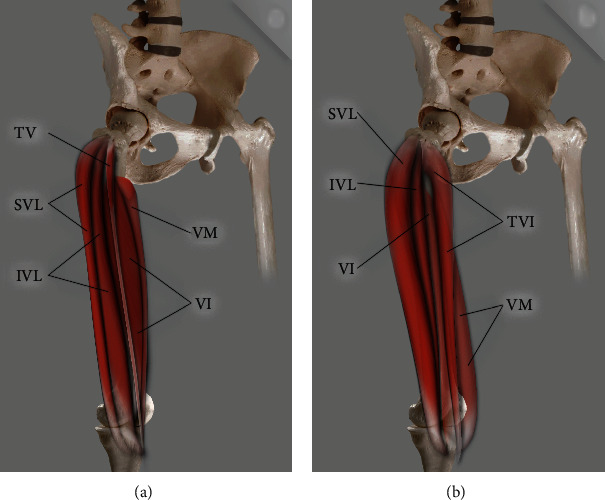
Scheme of type I quadriceps femoris. (a) Type IA quadriceps femoris: TVI: tensor vastus intermedius; SVL: superficial part of the vastus lateralis; IVL: intermediate part of the vastus lateralis; VI: vastus intermedius; VM: vastus medialis. (b) Type IB quadriceps femoris: TVI: tensor vastus intermedius; SVL: superficial part of the vastus lateralis; IVL: intermediate part of the vastus lateralis; VI: vastus intermedius; VM: vastus medialis.

**Figure 2:**
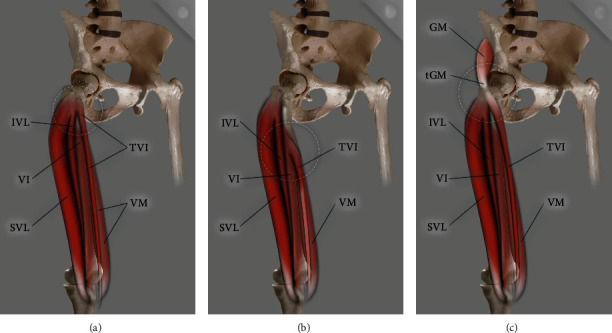
Scheme of type II quadriceps femoris. (a) Type IIA quadriceps femoris: TVI: tensor vastus intermedius; SVL: superficial part of the vastus lateralis; IVL: intermediate part of the vastus lateralis; VI: vastus intermedius, VM: vastus medialis. (b) Type IIB quadriceps femoris: TVI: tensor vastus intermedius; SVL: superficial part of the vastus lateralis; IVL: intermediate part of the vastus lateralis; VI: vastus intermedius; VM: vastus medialis. (c) Type IIC quadriceps femoris: TVI: tensor vastus intermedius; SVL: superficial part of the vastus lateralis; IVL: intermediate part of the vastus lateralis; GM: gluteus minimus; tGM: gluteus minimus tendon.

**Figure 3:**
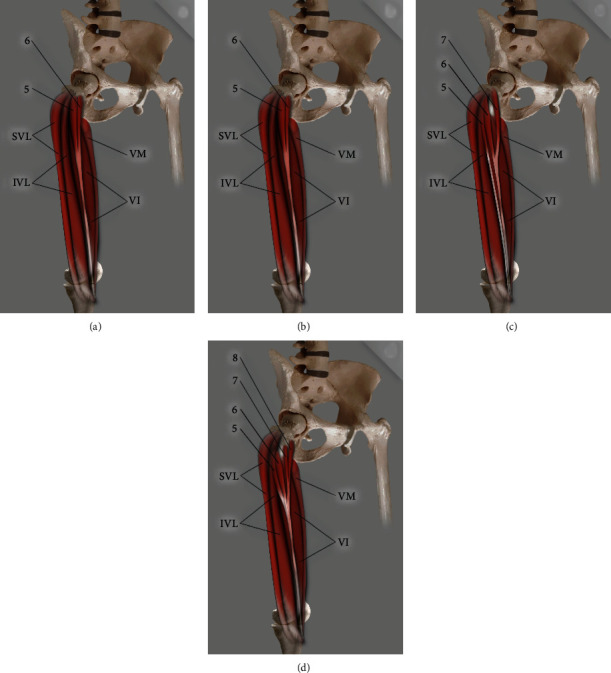
Scheme of Type III quadriceps femoris. (a) Type IIIA quadriceps femoris: 5: fifth head; 6: sixth head; SVL: superficial part of the vastus lateralis; IVL: intermediate part of the vastus lateralis; VI: vastus intermedius; VM: vastus medialis. (b) Type IIIB quadriceps femoris: 5: fifth head; 6: sixth head; SVL: superficial part of the vastus lateralis; IVL: intermediate part of the vastus lateralis; VI: vastus intermedius; VM: vastus medialis. (c) Type IIIC quadriceps femoris: 5: fifth head; 6: sixth head; SVL: superficial part of the vastus lateralis; IVL: intermediate part of the vastus lateralis; VI: vastus intermedius; VM: vastus medialis. (d) Type IIID quadriceps femoris: 5: fifth head; 6: sixth head; 7: seventh head; 8: eighth head; SVL: superficial part of the vastus lateralis; IVL: intermediate part of the vastus lateralis; VI: vastus intermedius; VM: vastus medialis.

The QF had one or more supplementary heads in 68 dissected limbs from 106 cadaver's limbs (64.1%). The additional heads were identified in accordance with the previous studies [7]. Three main types, with subtypes, were distinguished (Figures 1, 2, and 3; Table 1).

## 4. Vasti Muscles

**Figure 4:**
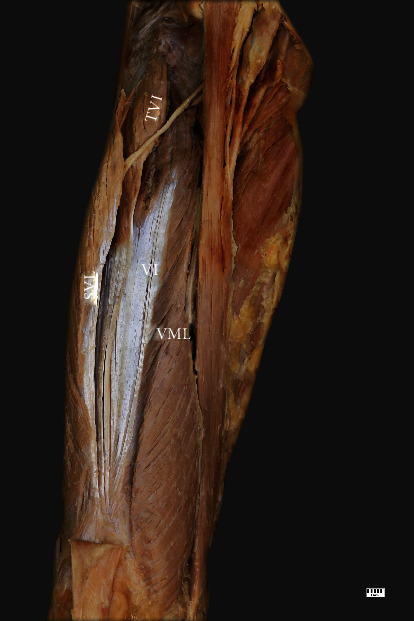
The multiceps femoris. Anterior view of the thigh. TVI: tensor vastus intermedius; SVL: superficial part of the vastus lateralis muscle; VI: vastus intermedius muscle; VML: vastus medialis longus.

**Figure 5:**
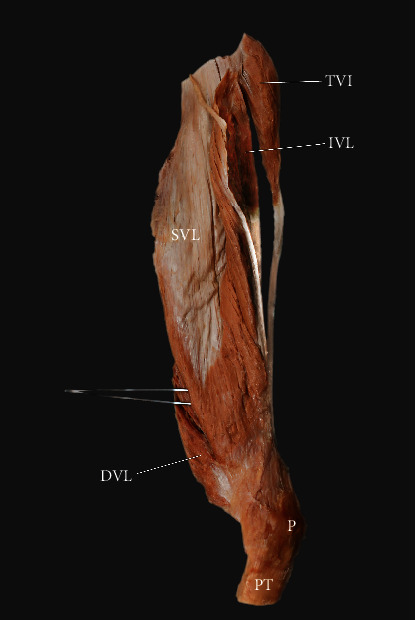
Extracted vastus lateralis from tensor vastus intermedius. Lateral view. SVL: superficial part of the vastus lateralis; IVL: intermediate part of the vastus lateralis; DVL: deep part of the vastus lateralis; P: patella; PT: patellar tendon; TVI: tensor vastus intermedius.

**Figure 6:**
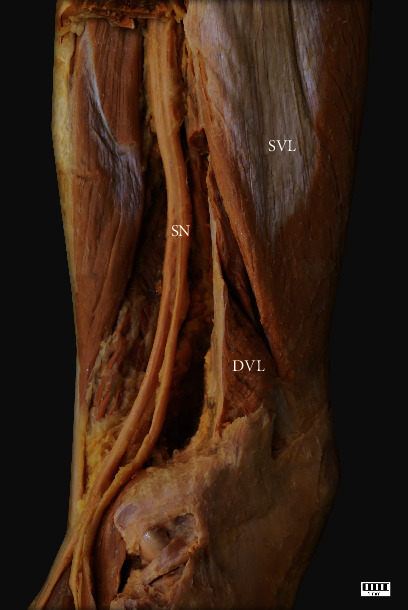
The vastus lateralis muscle. Posterolateral view of the thigh. SVL: superficial part of the vastus lateralis; DVL: deep part of the vastus lateralis; SN sciatic nerve.

The VL muscle consists of three layers: superficial, intermediate, and deep:

- The superficial part originates from the lateral surface of the greater trochanter. Its fibers arch medially downward to the tendon lamina and then to the QF tendon (Figures 4 and 5)
- The intermediate part originates from the upper level of the greater trochanter's anterior surface where it joins the intertrochanteric and gluteus medius ridge (Figures 4 and 5)
- The deep part is the one-third proximal to the femur (Figures 6 and 5)

**Figure 7:**
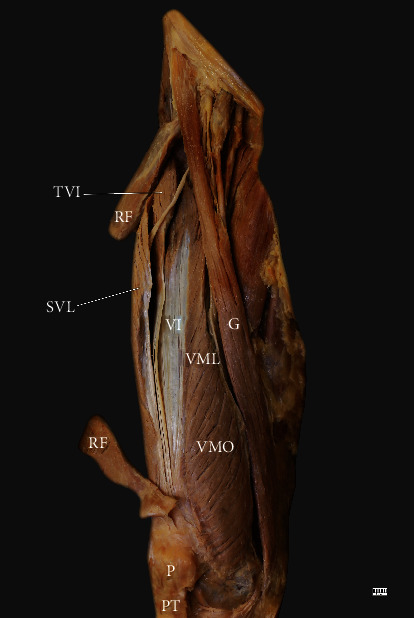
The multiceps femoris. Anterior view of the thigh. TVI: tensor vastus intermedius; RF: rectus femoris; SVL: superficial part of the vastus lateralis; VI: vastus intermedius; VML: vastus medialis longus; VMO: vastus medialis oblique; G: gracilis; P: patella; PT: patellar tendon.

The VM originates from the lower part of the intertrochanteric line, along the spiral line to the medial lip of the linea aspera, the medial intermuscular septum, and the aponeurosis of the adductor magnus, and forms the quadriceps tendon. The VM consists of the vastus medialis longus (VML) and vastus medialis obliquus (VMO) (Figure 7). While the fibers of the VML run anteroinferiorly, those of the VMO are more horizontal. There was no distinct fibrofascial plane or septum between the VML and VMO.

The VI originates from the upper two-thirds of the anterior and lateral surfaces of the femur and the intermuscular septum and forms the quadriceps tendon (Figure 4).

Differences in morphological parameters of vasti muscles between genders and body sides are presented in supplementary Tables 1a and 1b while between types of additional heads in supplementary Tables 2a and 2b. Most valuable difference that we observed was the following:

- The distal portion of the patella was wider in lower limbs without supplementary heads than in type IA but narrower than in type IIIA. In general, the distal portion of the patella was narrower in specimens with a supplementary head than in those without (19.03 SD 3.18 mm vs. 20.58 SD 2.95 mm, *p* = 0.03817). Other patellar ligament dimensions did not differ significantly

## 5. Discussion

In the present study, the morphologies of the VL, VM, and VI and their relationships to additional heads of the QF were examined. The correlation between the additional QF heads and the patellar tendon was determined.

In recent years, increasing numbers of studies have examined morphological variations in human arteries, veins, nerves, muscles, tendons, and ligaments [26, 29–33]. Morphological variations can have significant effects on nerve compression [34, 35] or artery or vein compression [36], and an accessory hepatic artery or collateral circulation can facilitate surgical procedures [32]. The most frequent morphological variations seem to be vascular and neural variations, followed by those of the musculoskeletal system [37].

While morphological variations can possibly be explained from an evolutionary perspective, it is first necessary to understand all possible variations. One example is the plantaris muscle, which was previously considered residual; but our research suggests that it can only be developed in humans [38–40], like the fibularis tertius muscle [41]. Complete understanding of all clinical and evolutionary aspects of the human body requires a full study of the morphological variability of the musculoskeletal and vascular organs.

Previous studies of the QF have tended to focus on the multilaminar nature of the vasti. The VL consists of three parts, referred to as superficial, intermediate, and deep [42, 43]. The present study confirms this complexity of the VL. There is some evidence that the VL contracts before the VM in patients with patellofemoral pain syndrome; this has been proposed as a cause of knee pain [44]. In addition, as each part is separately innervated, the muscle can be used for selective flap harvesting [43].

The VM comprises two parts, the VMO and VML [45]. Our present findings confirm this, though our analysis was only superficial. Patellar maltracking and patellofemoral pain are treated by strengthening the oblique fibers of the VM to restore the balance between the VM and VL and assessing of the degree of dynamic supination and pronation of the foot [7, 45].

Testut in 1884 first mentioned the morphological variability of QF, i.e., the lack of one or other part [46]. He described a triceps crural (femoris) muscle comprising the RF, VM, and VL [46]. The same observation was made by LeDouble in 1897 [2]. Bonnechere et al. [8] reported four cases of triceps femoris attributable to the absence of VI and VM or VI and VL.

**Figure 8:**
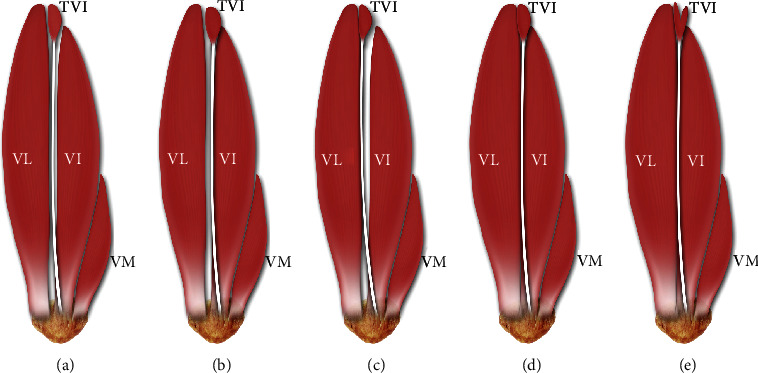
Types of TVI muscles according to Grob et al. [4]. (a) Independent type. (b) VI type. (c) VL type. (d) Common type. (e) Two muscle bellies type. P: patella; VL: vastus lateralis muscle; VI: vastus intermedius; VM: vastus medialis; TVI: tensor vastus intermedius.

In recent years, there has been much controversy about the presence of a fifth head of the QF. The first reports of VL or VM duplication were by Le Double [2] and MacAlister, while Golland et al. noted a fifth head of the QF between the VL and VI in 1986 [9]. Holyoke [47] described a *potential fifth head* as an aberrant belly. Interestingly, Grob et al. [4] classified a fifth head of the QF as the tensor vastus intermedius (TVI) and created a five-fold classification of it, based primarily on the course of the aponeurosis tendon. A detailed description of this classification is given in Table 2 and Figure 8.

This classification was confirmed in a study of 36 limbs of the South Indian population [10]; however, the tendons showed little diversity. Another study found a TVI in only seven of 20 limbs [8]; in addition, the VI and VM could not be differentiated, nor could the VI and VL.

Our recent research suggests that the Grob et al. classification [4] is not sufficient; however, an alternative based on proximal attachment has been proposed [7]. This second classification has three main divisions with subtypes: type I (subtypes A-B) includes a completely independent type TVI. Type IA originates from the upper level of the anterior surface of the greater trochanter, where it joins the intertrochanteric and gluteus medius ridge. The muscle belly runs laterally with respect to the VI. Type IB has the same proximal attachment as type IA, but the muscle belly runs medial to the VI. Type II consists of three subtypes based on the source of the TVI (A-C): type IIA from the VL, type IIB from the VI, and type IIC from the gluteus minimus. In contrast, type III has multiple heads; the number of subtypes depends on the number of additional muscles (six, seven, or eight). Detailed descriptions and percentages of occurrence are presented in Table 1.

The incidence of the fifth head ranges from 29% to 100% [4, 8, 10, 11]. Some cases have a separate VL muscle belly (29% of dissected limbs) [9], or an additional muscle can be found between the VL and VI (36% of cases) [11]. A TVI has also been found (35% of limbs) [8]. Other studies have identified a TVI in all limbs [4, 10]; Olewnik et al. [7] found it in 64.1% of all cases.

The effects of the presence or absence of the TVI are unknown, and whether it is a muscle that is still being developed is not clear, like the fibularis tertius [41] or plantaris [29, 38–40], or a vestigial muscle like the palmaris longus [35].

The muscle in the musculotendinous junction was significantly thicker in type IIIa than in all other types apart from type IB. This could possibly allow people with type IIIA and type IB to transfer a much greater contraction force from the muscle belly to the tendon, thus reducing forces on the tendons in other parts of the QF. To confirm this, complex biomechanical tests will be needed.

The tendon of the VI was significantly longer in types IIA and IIIA than in IB, IIB, or IIIB. Generally, muscles with a wide and short tendon are characterized by the transfer of high contraction forces from the belly to the tendon [33]. Future research must focus on comparing strengths among different types of extra heads.

The distal portion of the patella was wider in lower limbs without supplementary heads than in type IA but narrower than in type IIIA. In general, the distal portion of the patella was narrower in specimens with supplementary heads than in those without (19.03 SD 3.18 mm vs. 20.58 SD 2.95 mm, *p* = 0.03817). It is noteworthy that the patellar ligament is wider when additional QF heads are present, indicating that the ligament can transfer much greater forces over the knee joint than when there are no such heads. Are people with extra heads therefore less likely to suffer from loss of QF muscle strength or knee injury?

The TVI muscle is needed for knee extension, and its action can be supported because it originates from the VI or VL. It is possible that it can improve rehabilitation after ACL reconstructions by hastening QF recovery. Interestingly, extension of the gluteus minimus tendon, from which the TVI originates, can also affect the hip joint.

This study has some limitations. First, the study population was recruited from the specific populations of people who have lived the better part of their lives in the region around Lodz, Poland, and around Madrid, Spain. In addition, the deep layer of the VM part was not examined extensively. Finally, our statistical results require further biomechanical research. Our findings in this article have revealed nothing about the effect of additional quadriceps femoris heads on the vasti muscles and patellar ligaments.

## 6. Conclusion

The quadriceps femoris muscle is characterized by high morphological variability. Occurrence of extra heads is at the level of 64.1%. The vastus lateralis consists of three parts (superficial, intermediate, and deep), and vastus medialis consists of two (longus and oblique).

## Figures and Tables

**Table 1:** Classification of the proximal attachment by Olewnik et al. [7].

Type	Subtype	Description	Origin	Number/percentage
I	A	Single fifth head and independent muscle	Originates from the upper level of the greater trochanter's anterior surface where it joins the intertrochanteric and gluteus medius ridge. The muscle belly runs laterally to the VI.	20 (29.4%)
	B		Originates from the upper level of the greater trochanter's anterior surface where it joins the intertrochanteric and gluteus medius ridge; however, the muscle belly runs medial to the VI.	10 (14.7%)

II	A	The fifth head grows out from other muscles	From the VL.	16 (23.5%)
	B		From the VI.	3 (4.5%)
	C		From the gluteus medius.	2 (2.9%)

III	A	Multiple supplementary heads	Two heads with a single common tendon. The first (lateral head) originates from the upper level of the greater trochanter's anterior surface where it joins the intertrochanteric and gluteus medius ridge; the second (medial head) originates from the femur's anterior surface just above the VI muscle's proximal attachment.	4 (5.9%)
	B		Two heads with two separate tendons. The first head (lateral head) originates from the upper level of the greater trochanter's anterior surface where it joins the intertrochanteric and gluteus medius ridge; the second (medial head) originates from the femur's anteromedial surface just above the VI muscle proximal attachment.	10 (14.7%)
	C		Three heads (lateral, intermediate, and medial). The lateral and intermediate heads originate from the VL, while the medial head originates from the upper level of the greater trochanter's anterior surface where it joins the intertrochanteric and gluteus medius ridge. The intermediate and medial heads join and form a common tendon.	2 (2.9%)
	D		Four heads (bifurcated lateral and bifurcated medial). The bifurcated medial form consists of medial and lateral heads. The medial originates from the femur's innominate tubercle [28], and the lateral originates from the inferior level of the greater trochanter's anterior surface; these two heads join to form a common tendon. In addition, the bifurcated lateral form consists of medial and lateral heads: the medial originates from the inferior level of the greater trochanter's anterior surface and from the intermediate part of the VL; the lateral originates from the intermediate part of the VL and from the anterolateral surface of the shaft of the femur, lateral to the VI, and then the two heads join and form a common tendon.	1 (1.5%)

**Table 2:** Classification by Grob et al. [4].

Type	Description	Frequency of occurrence
Independent type	The muscle takes origin from the upper part of the intertrochanteric line and the anterior part of the greater trochanter, but the origin is separable from that of VL. The aponeurosis is separable from both VI and VL.	11 (42%)
VI type	The muscle takes origin together with VI, and the posterior border of TVI is fused with VI. The aponeurosis is separable from VL.	6 (23%)
VL type	The muscle takes origin together with VI, and the posterior border of TVI is fused with VI. The aponeurosis is separable from VL.	5 (19%)
Common type	The muscle takes origin from the VL, and the origin is inseparable. The aponeurosis is separable in both VL and VI.	4 (15%)
Two muscle bellies	The muscle is characterized by two muscle bellies and a converging tendon inserting into the patella.	5 (19%)

## Data Availability

Please contact author for data requests (Łukasz Olewnik PhD - email address: lukasz.olewnik@umed.lodz.pl).

## Supplementary Materials

Supplementary Materials Table 1a: morphometric parameters of vastus lateralis according to gender and body side. Table 1b: morphometric parameters of vastus medialis and intermedius and patellar ligament according to gender and body side. Table 2a: morphometric parameters of vastus lateralis according to main types of. Table 2b: morphometric parameters of vastus medialis and intermedius and patellar ligament according to gender and body side.
