# Supplementary material for: The relationship between additional heads of the quadriceps femoris, the vasti muscles, and the patellar ligament
Source: Biomed Res Int. 2022 Feb 16;2022:9569101. doi: 10.1155/2022/9569101 (PMC8866009; doi:10.1155/2022/9569101)
Supplement: Supplementary Materials — Table 1a: morphometric parameters of vastus lateralis according to gender and body side. Table 1b: morphometric parameters of vastus medialis and intermedius and patellar ligament according to gender and body side. Table 2a: morphometric parameters of vastus lateralis according to main types of. Table 2b: morphometric parameters of vastus medialis and intermedius and patellar ligament according to gender and body side. [file 9569101.f1.docx]

Table 1a. Morphometric parameters of vastus lateralis according to gender and bodyside.

|  | Superficial part | | | | | | | Intermediate part | | | | | | Deep part | | | | | |
| --- | --- | --- | --- | --- | --- | --- | --- | --- | --- | --- | --- | --- | --- | --- | --- | --- | --- | --- | --- |
|  | Muscle belly | | | | M-T junction | | Tendon length | Muscle belly | | | M-T junction | | Tendon length | Muscle belly | | | M-T junction | | |
|  | L | Apo | ProW | ProT | W | T |  | L | W | T | W | T |  | L | W | T | W | T | L |
| Female | 272.78 (45.12) | 214.32 (31.70) | 25.26 (7.19) | 4.18 (1.66) | 25.04 (8.39) | 3.52 (1.78) | 42.25 (21.35) | 124.33 (41.80) | 19.87 (6.58) | 4.66 (2.69) | 19.12 (7.10) | 1.90 (1.49) | 188.92 (36.45) | 143.18 (26.36) | 14.26 (4.86) | 3.73 (1.87) | 15.10 (5.92) | 3.84 (1.52) | 32.14 (17.53) |
| Male | 294.24 (35.84) | 209.45 (43.11) | 25.43 (6.26) | 3.68 (1.76) | 27.31 (9.93) | 3.32 (1.81) | 44.64 (20.85) | 136.27 (40.08) | 27.47 (14.55) | 4.43 (2.14) | 25.07 (11.66) | 2.50 (2.06) | 195.98 (48.00) | 133.70 (35.17) | 13.22 (4.24) | 3.20 (1.81) | 12.54 (5.70) | 3.04 (1.73) | 37.64 (18.48) |
| P | 0.9024 | 0.1676 | 0.6914 | 0.0026 | 0.1624 | 0.2164 | 0.6161 | 0.4163 | 0.9054 | 0.1428 | 0.0056 | 0.9842 | 0.9960 | 0.9329 | 0.1612 | 0.0068 | 0.9130 | 0.7778 | 0.0041 |
| Right | 287.02 (37.72) | 209.33 (42.22) | 25.15 (7.00) | 3.88 (1.79) | 26.53 (9.58) | 3.41 (1.75) | 42.73 (17.18) | 132.59 (41.20) | 24.40 (12.37) | 4.45 (2.37) | 23.22 (10.36) | 2.17 (1.71) | 194.63 (43.46) | 183.48 (43.65) | 139.64 (33.00) | 12.94 (4.20) | 3.41 (1.81) | 12.79 (5.85) | 3.34 (1.61) |
| Left | 285.30 (43.95) | 212.49 (37.87) | 25.59 (6.20) | 3.85 (1.69) | 26.33 (9.29) | 3.37 (1.85) | 44.83 (24.35) | 131.15 (41.05) | 25.05 (13.31) | 4.58 (2.32) | 22.65 (10.98) | 2.39 (2.06) | 192.07 (44.97) | 176.25 (35.84) | 134.53 (32.10) | 14.28 (4.70) | 3.36 (1.89) | 14.10 (5.90) | 3.31 (1.79) |
| P | 0.8327 | 0.7651 | 0.7463 | 0.9395 | 0.9138 | 0.9131 | 0.6196 | 0.8628 | 0.8051 | 0.7971 | 0.7955 | 0.5743 | 0.7764 | 0.4385 | 0.4744 | 0.1803 | 0.9027 | 0.3404 | 0.9236 |

Significant p according to Bonferroni correction is < 0.001; L – length. Apo – aponeurosis. Pro – proximal. W – width. T – thickness.

Table 1b. Morphometric parameters of vastus medialis and intermedius. and patellar ligament according to gender and bodyside.

| Type | Vastus intermedius | | | Vastus medialis | | | | | | Patellar ligament | | | | | | |
| --- | --- | --- | --- | --- | --- | --- | --- | --- | --- | --- | --- | --- | --- | --- | --- | --- |
|  | Muscle belly | | Tendon length | Muscle belly | | | M-T junction | | Tendon length | L | Prox. | | Mid. | | Dis. | |
|  | L | Apo |  | L | W | T | W | T |  |  | W | T | W | T | W | T |
| Female | 227.55 (57.80) | 123.93 (65.65) | 81.77 (62.70) | 283.87 (24.95) | 14.30 (5.72) | 2.85 (1.51) | 30.67 (9.50) | 4.38 (3.93) | 20.84 (14.92) | 51.22 (12.50) | 26.32 (3.94) | 3.22 (1.18) | 21.52 (2.32) | 3.67 (1.02) | 19.12 (3.03) | 2.60 (1.07) |
| Male | 230.51 (53.44) | 155.46 (20.17) | 72.19 (47.63) | 303.00 (34.99) | 13.90 (6.09) | 3.53 (1.47) | 29.14 (10.13) | 3.79 (3.44) | 22.22 (13.03) | 56.61 (13.18) | 26.37 (5.11) | 4.20 (1.60) | 21.53 (3.34) | 3.90 (1.50) | 19.95 (3.26) | 3.37 (1.07) |
| P | 0.0242 | 0.2784 | 0.2404 | 0.6697 | 0.1486 | 0.0287 | 0.1615 | 0.0146 | 0.9249 | 0.1141 | 0.2489 | 0.6440 | 0.1929 | 0.0994 | 0.0323 | 0.0427 |
| Right | 227.65 (53.94) | 154.83 (29.12) | 74.04 (53.98) | 294.73 (31.97) | 14.00 (5.82) | 3.31 (1.49) | 29.24 (8.84) | 3.73 (3.25) | 21.89 (14.80) | 54.23 (12.33) | 26.19 (4.70) | 3.74 (1.44) | 21.52 (2.94) | 3.75 (1.22) | 19.61 (3.05) | 2.98 (1.12) |
| Left | 231.19 (56.25) | 142.13 (43.81) | 77.08 (53.06) | 296.44 (33.75) | 14.08 (6.14) | 3.28 (1.55) | 30.25 (10.87) | 4.31 (3.99) | 21.38 (12.86) | 54.35 (14.00) | 26.51 (4.59) | 3.82 (1.59) | 21.53 (2.95) | 3.85 (1.42) | 19.56 (3.33) | 3.09 (1.15) |
| P | 0.7472 | 0.3931 | 0.7804 | 0.7982 | 0.9492 | 0.9174 | 0.6466 | 0.4718 | 0.8670 | 0.9674 | 0.7630 | 0.8000 | 0.9899 | 0.7326 | 0.9395 | 0.6937 |

Significant p according to Bonferroni correction is < 0.001; L – length. Apo – aponeurosis. Pro – proximal. W – width. T – thickness.

Table 2a. Morphometric parameters of vastus lateralis according to main types of .

| Type | Superficial part | | | | | | | Intermediate part | | | | | | Deep part | | | | | |
| --- | --- | --- | --- | --- | --- | --- | --- | --- | --- | --- | --- | --- | --- | --- | --- | --- | --- | --- | --- |
|  | Muscle belly | | | | M-T junction | | Tendon length | Muscle belly | | | M-T junction | | Tendon length | Muscle belly | | | M-T junction | | |
|  | L | Apo | ProW | ProT | W | T |  | L | W | T | W | T |  | L | W | T | W | T | L |
| 1 | 296.43 (24.07) | 219.39 (27.96) | 27.26 (5.99) | 3.66 (1.52) | 30.46 (8.67) | 3.17 (1.26) | 36.53 (17.69) | 135.92 (30.24) | 24.02 (10.62) | 5.08 (2.62) | 22.78 (8.17) | 3.16 (2.29) | 183.52 (36.49) | 142.86 (30.07) | 13.75 (3.67) | 3.84 (1.77) | 11.63 (3.84) | 3.93 (2.02) | 28.73 (11.01) |
| 2 | 288.53 (31.71) | 223.97 (16.40) | 27.81 (6.69) | 4.49 (2.07) | 22.75 (8.88) | 3.69 (1.98) | 49.88 (11.33) | 123.60 (48.08) | 22.99 (14.31) | 3.74 (2.37) | 25.27 (14.76) | 1.73 (1.57) | 193.18 (43.71) | 139.07 (31.08) | 14.44 (4.44) | 4.46 (1.67) | 16.01 (7.67) | 3.29 (1.46) | 38.96 (16.27) |
| 3 | 290.20 (19.38) | 208.68 (49.49) | 24.39 (3.75) | 4.08 (2.04) | 23.66 (8.55) | 4.48 (2.68) | 48.42 (26.67) | 127.49 (28.68) | 22.39 (9.66) | 4.31 (1.51) | 19.13 (11.69) | 2.29 (2.26) | 194.62 (33.63) | 146.01 (28.94) | 12.70 (3.93) | 2.74 (1.45) | 16.75 (3.47) | 2.61 (1.45) | 35.90 (15.31) |
| P | 0.1501 | 0.5155 | 0.0059 | 0.3592 | 0.0148 | 0.2254 | 0.0219 | 0.5426 | 0.4536 | 0.2944 | 0.3298 | 0.0083 | 0.2394 | 0.3669 | 0.7304 | 0.0006 | 0.0239 | 0.1939 | 0.1228 |

Significant p according to Bonferroni correction is < 0.0025; L – length, Apo – aponeurosis, Pro – proximal, W – width, T – thickness.

Table 2b. Morphometric parameters of vastus medialis and intermedius. and patellar ligament according to gender and bodyside.

| Type | Vastus intermedius | | | Vastus medialis | | | | | | Patellar ligament | | | | | | |
| --- | --- | --- | --- | --- | --- | --- | --- | --- | --- | --- | --- | --- | --- | --- | --- | --- |
|  | Muscle belly | | Tendon length | Muscle belly | | | M-T junction | | Tendon length | L | Prox. | | Mid. | | Dis. | |
|  | L | Apo |  | L | W | T | W | T |  |  | W | T | W | T | W | T |
| 1 | 238.13 (43.64) | 154.53 (22.49) | 60.05 (26.60) | 300.88 (27.53) | 11.02 (4.36) | 3.58 (1.52) | 27.38 (10.02) | 4.82 (4.28) | 21.58 (15.06) | 49.84 (9.77) | 25.17 (4.28) | 3.68 (1.37) | 20.91 (2.92) | 3.72 (1.09) | 18.05 (2.98) | 3.07 (0.90) |
| 2 | 232.87 (74.92) | 174.72 (0.70) | 94.04 (86.35) | 286.12 (29.03) | 15.02 (5.99) | 2.46 (1.07) | 31.64 (8.68) | 4.00 (3.60) | 25.86 (11.34) | 53.74 (15.21) | 26.87 (5.07) | 3.54 (1.56) | 21.31 (2.58) | 3.48 (0.89) | 18.65 (2.10) | 2.59 (1.37) |
| 3 | 211.60 (52.27) | 136.74 (25.00) | 77.68 (65.22) | 295.94 (36.11) | 18.09 (6.86) | 3.12 (1.17) | 30.54 (11.61) | 3.02 (1.47) | 16.61 (7.10) | 71.31 (13.78) | 29.32 (4.30) | 5.64 (1.55) | 23.64 (3.31) | 5.63 (1.62) | 23.00 (2.78) | 3.76 (1.28) |
| P | 0.1741 | 0.2229 | 0.3997 | 0.5028 | 0.0036 | 0.0363 | 0.0798 | 0.4240 | 0.4214 | 0.0031 | 0.1733 | 0.0105 | 0.2582 | 0.0130 | 0.0001 | 0.2269 |

Significant p according to Bonferroni correction is < 0.0031; L – length, Apo – aponeurosis, Pro – proximal, W – width, T – thickness.
